# Entropion Cured by Pulling down the Eyelid

**Published:** 1829

**Authors:** 


29. *Entropion cured by pulling down the eyelid.*—Dr. Jackson of Northumberland, has published in the Am. Jour. a case of entropion of one of the lower eyelids, consequent on long continued, miasmatic ophthalmia, which was suddenly cured by strongly pulling down the lid, in the manner proposed by Demours. The following paragraphs will sufficiently set forth the condition of the eye and the method which he employed:—

One eye was now entirely well, but the other was found to be affected with entropion of the lower palpebra. The whole tarsus was turned under the eyeball, so as to present a smooth rounded contour, as represented in M. Demours' eighteenth plate. We made several attempts to pull down the lid and extricate the tarsus, but so violent was the action of the muscles that we utterly failed; therefore, as the eye was constantly improving we desisted from any further attempt, with the hope that the new cilia had not grown out so as to cause irritation. The swelling diminished every day under the use of blisters; the globe was not inflamed, but some matter filled the eye in the morning; every blister most evidently lessened the sensibility to light.

Three weeks ago we made a very vigorous attempt, with Dr. Rodrigue's help to pull out the tarsus, but succeeded only so far as to discover that the cilia had grown to nearly their full length and full number, and that the eyeball, rolling upon them, held the tarsus firmly under. Here was at once an end to all our dreams of a cure; for on such a restive child, now rendered infinitely impatient of every thing, we considered an operation as impracticable, and that the cilia would irritate the eye afresh, and prove a cause of insuperable inflammation. Wishing however to explore the state of things yet further, we made another violent attempt to pull out the tarsus, and fortunately the little sufferer did not resist, as she had always done before, with the utmost power of the orbicularis; so far from it, that, as it were by inspiration, she seized the palbebra with all her fingers, while mine were employed lower down, and she turned the tarsus completely out so that the cilia spread over the front of the eye. It appeared to have no tendeacy to relapse; I saw it resume its natural situation and figure, nor could I compare the operation to any thing more appropriately, than to the reduction of a dislocated bone. The eye and palpebra assumed their natural appearance at once, and have ever since continued entirely well. The cilia stood erect at first, and spread over the eye, but they gradually assumed their proper direction.

This case was clearly not produced by ulceration, and consequent contraction of the inside of the palpebra; we should rather presume that the lid had been so distorted by tumefaction, that the tarsus was reverted under the eye-ball, and afterwards firmly held there by the pressnre and rolling of the globe on the new cilia. This accords with the obseivations of M. Demours, that "the entropion of the inferior palpebra is most frequently the consequence of an œdema of its tissues, and particularly of a relaxation of that part of the skin which covers it."*

*Tome premier, p. 105, De la Trichaise.

